# Concurrent (Dual) Disorder Management Guidelines: Systematic Review Update

**DOI:** 10.3390/jcm15083123

**Published:** 2026-04-20

**Authors:** Syune Hakobyan, Zachary Allan, Stephen Lee-Cheong, Kristina Adorjan, Peter Falkai, Christian G. Schütz

**Affiliations:** 1 Department of Psychiatry, The University of British Columbia, Vancouver, BC V6T 1Z4, Canada; zack.allan@ubc.ca (Z.A.);; 2 Department of Psychiatry, University of Bern, 3008 Bern, Switzerland; 3 Department of Psychiatry, Ludwig Maximilians University of Munich, 80539 Munich, Germany; 4 British Columbia Mental Health and Substance Use Research Institute, Provincial Health Service Authority, Vancouver, BC V5C 6E3, Canada

**Keywords:** concurrent disorder, co-occurring disorder, dual diagnosis, dual pathology, addiction comorbidity, comorbid substance abuse, comorbid illicit use, comorbid addiction, comorbid mental illness, coexisting mental illness

## Abstract

**Background/Objectives:** The initial systematic review of “Concurrent Disorder Management Guidelines. Systematic Review” assessed the quality of the concurrent disorders’ clinical management guidelines in 2020, including the guidelines in the field from 2000 to 2020. Twenty-four guidelines were identified and assessed with AGREE II (Appraisal of Guidelines for Research and Evaluation). As dual disorder needs increased specifically among the younger population, requiring significant healthcare resources, more efficient approaches targeting complex concurrent disorders are essential. Since 2020, multiple new guidelines have been developed in response to new developments in the field of substance use disorder management. This systematic review update aimed to identify and appraise all new available concurrent disorder management guidelines to strategize the management of concurrent disorders, support better outcomes and further research directions. **Methods:** The review was registered, and protocol is available in the international register—PROSPERO. Literature searches were performed by two independent authors in electronic databases and the gray literature. The inclusion criteria were English language clinical management guidelines for adult concurrent disorders between 2020 and 2025. Sources that were not formal clinical guidelines, not addressed to physicians for adult age group, addressed to intellectual/developmental disability, or written in languages other than English were excluded. **Results:** The initial search resulted in 5003 records. A total of eight new guidelines were identified and assessed with AGREE II, highlighting the consistent gap in the evidence-based management recommendations. **Conclusions:** The appraised guidelines had similar quality to the 2020 findings, supporting dual or combined treatment; however, all guidelines had multiple domains not developed rigorously and with methodological limitations. Levels of complexity and staging of treatment were not considered in recommendations. Average domain scores were very low, with the lowest being applicability and editorial independence. Development of high-quality, rigorously developed, evidence-based guidelines, addressing staging, resource implications, and patient involvement is recommended as the evidence base remains underdeveloped.

## 1. Introduction

In 2020 our “Concurrent Disorder Management Guidelines. Systematic Review” was published, addressing concurrent disorder (co-occurring disorder, dual diagnosis, comorbidity, and multiple other terminologies addressing the same or very similar issue) guidelines, which refers to a specific form of mental health multimorbidity where at least one substance use disorder and at least one non-substance-bound mental disorder are in need of treatment simultaneously [1,2,3,4]. The original review did not include developmental and intellectual disabilities with mental health concerns that are considered as a “dual diagnosis” or “concurrent disorder”.

As people with concurrent disorders are at risk of being underdiagnosed, undertreated, and experience high burden of morbidity and mortality, practicing best available management recommendations can mitigate the mentioned risks. The initial purpose of the study was to systematically review the most current management guidelines available and explore the scope, approach, structure, knowledge limitations, and consistency to make suggestions for future development. The original study identified 24 guidelines in total and were assessed using the guideline assessment standardized tool—AGREE II (Appraisal of Guidelines for Research and Evaluation). It revealed that most guidelines’ standards were acceptable; however, only the NICE guidelines had all detailed information on all AGREE II domains [1,5]. Combinations of treatments for individual disorders were generally supported by guidelines, with a very small evidence base for concurrent disorders, and they provided little recommendation for further structuring of the field, such as level of complexity or staging, or evaluating different models of treatment integration.

Concurrent disorders remain a major global clinical and public health challenge. Individuals with co-occurring mental health and substance use disorders experience elevated morbidity and mortality, poorer treatment outcomes, and increased healthcare utilization compared with individuals with single diagnoses [6,7,8]. As the burden of concurrent disorders continues to grow worldwide, more effective and integrated approaches for managing complex dual pathology are increasingly needed [6,7,8,9]. Six years after the last guideline update, concurrent disorders are now more firmly recognized as the default clinical expectation rather than an expectation. Since the last paper was published in 2020, the prevalence of concurrent disorders in the United States among adults aged 18 years or older has increased from 5.7 million people to 6.9 million in 2024 [10,11]. While the prevalence and clinical impact of concurrent disorders are well established, healthcare systems and service models have adapted very slowly. Data from 2024 suggest that, within the past year, 41.2% of people with concurrent disorders did not receive treatment for either substance use or mental health conditions [12].

Similar to our previous publication, this paper aims to inform future management recommendations that are currently being developed. Advances over the past six years in transdiagnostic dimensional models (that are neurobiologically informed) and the accumulation of evidence supporting the integration and psychological and pharmacological treatment rather than traditional sequential treatment models both warrant integration into updated guidelines [13,14,15].

The traditional categorical diagnostic models for mental health and disorders fail to capture the multidimensional, neurobiological, developmental, and environmental complexity of concurrent disorders, and do not align with the emergence of personalized medicine as a central principle in psychiatry [16,17,18]. Within an updated guideline framework, we hope to incorporate stratified and personalized decision-making that is aligned with contemporary evidence and clinical realities.

This systematic review aims to update and systematically appraise new published guidelines after 18 March 2020, since the first published systematic review, addressing the management of concurrent disorders in adult populations. The goal was to follow the original systematic review protocol and focus on concurrent disorder guidelines in the English language for healthcare professionals in primary, secondary, and tertiary care, but not to patients and their families, guidelines for persons with neurodevelopmental disorders and/or intellectual/developmental disabilities occurring simultaneously with concurrent disorder. The updated objective is to explore guidelines’ overall scope, approach, structure, knowledge limitations, consistency, methodological issues, potential bias issues, or other potential issues.

## 2. Materials and Methods

The protocol for this systematic review was prepared according to the PRISMA-P checklist [19,20], and the review was reported in accordance with PRIMSA 2000 guidelines [21]. The review was registered in the international register—PROSPERO (International Prospective Register of Ongoing Systematic Reviews, http://www.crd.york.ac.uk/prospero (accessed on 18 June 2025). The protocol of the study has been uploaded to PROSPERO (http://www.crd.york.ac.uk/prospero (accessed on 18 June 2025); ID: CRD420251076197) [22].

To identify novel guidelines in the field, two independent reviewers, S.H. and Z.A, completed literature searches and subsequent appraisals. When there was a disagreement, S.L.C. was involved, and, if any discrepancy, C.G.S. advised for a final decision. Reviewers have completed medical or psychology training and have experience working with individuals with concurrent disorders. The following electronic databases and websites were used to extract guidelines: MEDLINE (via Ovid), EMBASE (via Ovid), CINAHL, PsycINFO, JouleCMA, Trip, DynaMed, NICE Guidelines, SIGN, CADTH, and UpToDate. Additionally, a structured gray literature search was conducted through organizational websites relevant to concurrent disorders, including the Substance Abuse and Mental Health Services Administration (SAMHSAs), the Center for Addiction and Mental Health (CAMH) and the Canadian Network for Mood and Anxiety Treatments (CANMATs). Supplementary searches were performed using Google Scholar. Clinicians and researchers in the field were contacted in search of known information about guidelines in the field. All searches were set between 18 March 2020 and 18 June 2025. Search terms and key words are attached in the Supplementary File. The inclusion criteria included all published and unpublished English language clinical management guidelines of concurrent disorders that were published by professional organizations (e.g., American Psychiatric Association, World Health Organization, etc.), developed through systematic evidence review, expert consensus, formal approval process, and serve as authoritative standards of care.

We included formal concurrent disorders clinical management guidelines for appraisal using the AGREE (Appraisal of Guidelines for Research and Evaluation) II tool. The original AGREE instrument was developed to address the issue of variability in guideline quality, which assesses the methodological rigor and transparency of guideline development, and has since been refined to AGREE II [5]. We did not include sources from UpToDate or DynaMed for appraisal as these resources are not considered to be formal guidelines, while they are evidence-based. Several articles had information about management guidelines; however, this was a secondary focus, so they were not included for appraisal. Similarly, guidelines that were addressed to patients and their families, all relevant professionals, were considered for the review, while they were not appraised with the AGREE II. For the purposes of this review, developmental/intellectual disabilities occurring simultaneously with mental health concerns, also labeled “dual diagnosis” or “concurrent disorder”, were not considered [23,24]. Accordingly, the exclusion criteria were as follows: general reviews of concurrent disorder management, the literature that was not a formal guideline (although some of the guidelines were not classic formal guidelines and had focused review of available evidence as evidence base for the recommendations), non-English guidelines, the literature without concurrent disorders as their primary focus, the literature addressing persons with intellectual/developmental disabilities and concurrent mental disorders, and the literature that was published prior to 18 March 2020.

**Figure 1:**
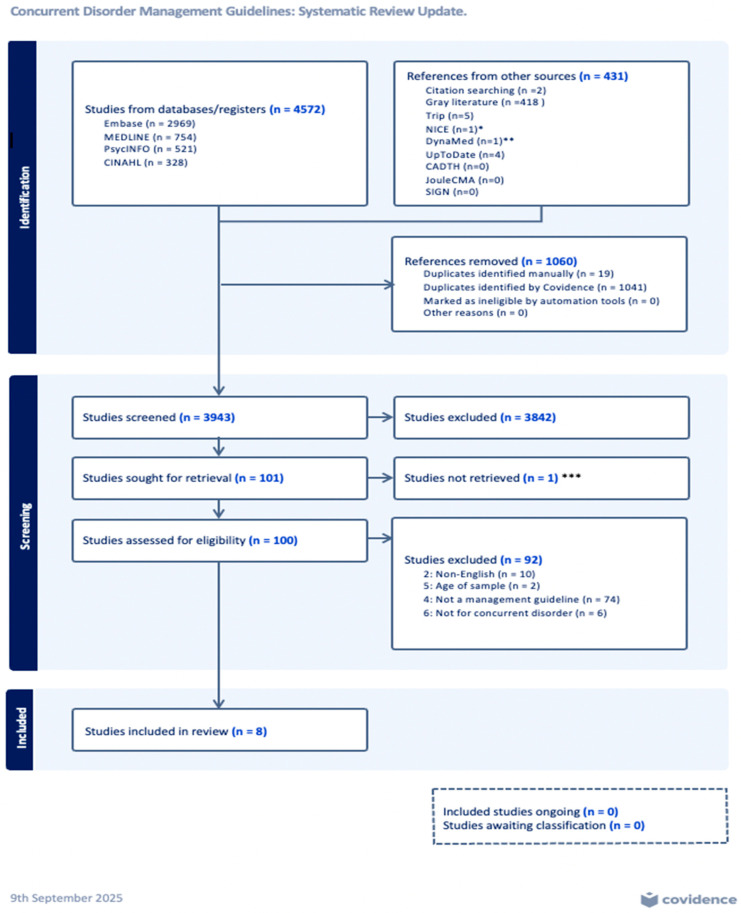
PRISMA Flow Diagram: retrieved from Covidence. * Coexisting severe mental illness and substance misuse: community health and social care services, Published 30 November 2016 (A presentational change was made on 14 August 2024. There were no changes to practice). ** “Co-occurring Substance Use Disorder and Mental Health Disorder” https://www.dynamed.com/condition/co-occurring-substance-use-disorder-and-mental-health-disorder-1 (accessed on 28 March 2026). *** Not available.

The final search revealed a total of 5003 results, comprising 4572 from an electronic database search and 431 from a gray literature search. The 4572 results from the electronic database search were all imported into Covidence (Veritas Health Innovation, Melbourne, Australia; available at: https://www.covidence.org (accessed on 28 March 2026)), which is a systematic review tool to import studies, screen titles, abstracts, papers, and compare the results between the reviewers [25]. Covidence automatically identified 1041 duplicates; 19 duplicates were identified manually, all of which were deleted. A total of 3512 articles remained in Covidence. The results of the gray literature and website search from different sources, including checking for updates on the appraised 24 papers from the original review (in total 431 results), were not uploaded to Covidence. Whenever possible, the removal of duplicate results in the gray literature search was done manually and assessed with the same approach. From both sources, the electronic database and gray literature/website searches, the study titles, abstracts, and full papers were examined by two reviewers (S.H. and Z.A.) to identify eligible studies based on the described inclusion criteria. Decisions of the two authors were recorded separately and in case of disagreement, they were discussed. In the absence of consensus, a decision was made by the third reviewer (S.L.C.), and finally, by the supervisory author (C.G.S.). During the title and abstract screening, independent reviewers achieved substantial agreement, with over 90% of decisions consistent between reviewers. All titles were scanned (3943) and if relevant to concurrent disorders, abstracts were read. Studies were classified for inclusion to appraise into YES (13), MAYBE (88) and NO (3842) groups. Electronic database search results were manually sorted within Covidence, while the gray literature and website results were manually sorted outside of it. In the YES and MAYBE groups, 100 full papers (80 from an electronic database + 20 from the gray literature and websites ***) were read. A full-text review was performed for the 100 selected studies and recorded in Covidence or in a study selection form, documenting the reason for the inclusion and exclusion of each study. After this process, 13 papers that fulfilled criteria remained and were subsequently considered for qualitative analysis. After full assessment, only eight papers fully fulfilled the inclusion criteria and were included in the final appraisal (Figure 1: PRISMA Flow Diagram 1). The AGREE II domain scores are presented as mean values across reviewers.

## 3. Results

### 3.1. Included to Appraise Guidelines

In total, eight clinical guidelines developed for concurrent disorders, published after the initial review, were included in the final appraisal analysis by AGREE II (Table 1).

Individual domain scores and overall quality of guidelines are presented in Table 2 and Table 3. The quality of different domains of guidelines had significant variability. The Australian government-developed “Guidelines on the Management of Co-Occurring Alcohol and other Drug and Mental Health Conditions in Alcohol and other Drug Treatment Settings” scored the highest of all guidelines across most domains, with very clear scope, purpose, and clear presentation. In contrast, “Principles of Care for Young Adults with Co-Occurring Psychiatric and Substance Use Disorders”, “SAMHSA Substance Use Disorder Treatment for People with Co-Occurring Disorders”, and “Psychological Treatment of PTSD with Comorbid Substance Use Disorder (SUD): Expert Recommendations of the European Society for Traumatic Stress Studies (ESTSS)” scored the lowest in AGREE II.

Almost all guidelines clearly described their objectives, purpose and scope; however, they did not contain all the required details about specific patient population, clear health intents, and expected outcomes. In “Management of Schizophrenia and Comorbid Substance Use Disorders: Expert Review and Guidance” description of population was poor.

The scores were low for stakeholder involvement from different groups representing the range of views and preferences of all target groups for many guidelines; however, the “TIP 42” by Substance Abuse and Mental Health Services Administration (SAMHSA), USA National Organization scored strongly. In contrast, “Management of Schizophrenia and Comorbid Substance Use disorder,” published by researchers from Spain, did not present comprehensive involvement of other stakeholders. A common concern was that none of the guidelines explicitly demonstrated preferences and views of the target population (public, patients, etc.). The guideline “TIP 42” described and defined the target users of their guidelines in a detailed manner.

Regarding rigor of development, both “Canadian Network for Mood and Anxiety Treatments (CANMAT) Task Force Report: A Systematic Review and Recommendations of Cannabis use in Bipolar Disorder and Major Depressive Disorder” and “Treatment Considerations for Youth and Young Adults with Serious Emotional Disturbances and Serious Mental Illnesses and Co-occurring Substance Use” had rigorous methodologies, and multiple resources to support their recommendations, but other guidelines presented significant weaknesses in the rigor to be able to comply with the required standards for developing evidence-based guidelines. External reviewing as part of current standards was not clearly completed in any guideline, despite multiple stakeholders being involved in development. Current standards also require guidelines to be revised regularly for providing up to date support, while the information related to the guideline updates was not available.

The requirements for current guidelines are that they need to clearly present important information. “TIP 42” had very clear details about the recommendations; however, some details in key recommendations were often absent. “Canadian Network for Mood and Anxiety Treatments (CANMAT) Task Force Report: A Systematic Review and Recommendations of Cannabis use in Bipolar Disorder and Major Depressive Disorder”, “Treatment Considerations for Youth and Young Adults with Serious Emotional Disturbances and Serious Mental Illnesses and Co-occurring Substance Use”, and “Guidelines on the Management of Co-Occurring Alcohol and other Drug and Mental Health Conditions in Alcohol and other Drug Treatment Settings” also scored very high.

Applicability constituted the weakest domain with deficiencies in most guidelines: issues such as resource implications were only discussed in “TIP42”, but issues of monitoring or auditing were not discussed in any guideline. Most were weak in presenting monitoring or auditing criteria with only “Guidelines Treatment Considerations for Youth and Young Adults with Serious Emotional Disturbances and Serious Mental Illnesses and Co-occurring Substance Use” and “Guidelines on the Management of Co-Occurring Alcohol and other Drug and Mental Health Conditions in Alcohol and other Drug Treatment Settings” addressing this adequately. Effective management often requires financial and logistical implementation to provide integrated service models, multidisciplinary teams, and coordination between mental health and addiction services. These models require substantial investments, including workforce training and infrastructure for care delivery. Future guidelines development would benefit from incorporating frameworks that explicitly address economic considerations, service capacity, and organizational barriers to adopting new practices.

Lastly, the information relevant to the editorial independence was not clearly mentioned in many guidelines. Guidelines did not provide information related to this important aspect of guideline development and did not address the guideline development group members’ competing interests.

“Management of Schizophrenia and Comorbid Substance Use Disorders: Expert Review and Guidance” was developed by Spanish researchers from university hospitals and research institutes. The objectives and health questions covered by the guideline were described well, targeting people with schizophrenia and substance use disorders; however, the specifics of population were not described in detail. It scored low on stakeholder involvement and rigor of development, while clear recommendations of who can use guidelines were also missing. Despite very comprehensive search, the domain development was not rigorously developed. The information presented was not very clear for clinical practice. The implication for resources to apply the recommendations was also limited. Lastly, the guideline did not have sufficient information on editorial independence. Overall, the quality was low, recommending use only with major modifications.

“Principles of Care for Young Adults with Co-Occurring Psychiatric and Substance Use Disorders”, which was published by the researchers from Boston University, Boston Medical Center, and University of California, was targeted on people with comorbid substance use and mental health disorders. The overall objectives, health question, and population to apply a guideline were well described. Although different groups were involved in development, patient preferences and information on the target users of the guideline were not described. Recommendations were clearly connected to evidence; however, the rigor of development and other components had low scores in AGREE II standards. On domains, presentations, applicability, and editorial independence, the scores were better; however, there were multiple areas that could be improved. Overall, recommendations are to be used only after significant changes to rigor of development.

“Guidelines on The Management of Co-Occurring Alcohol and Other Drug and Mental Health Conditions in Alcohol and Other Drug Treatment Settings. Treatment Improvement Protocol TIP 42” developed by Substance Abuse and Mental Health Services Administration, which is a branch of the U.S. Department of Health and Human Services and focuses on patients with co-occurring substance use disorders and mental health disorders. The scope and purpose of the guideline were well described, except for some minor information. The list of stakeholder involvement was impressively extensive, although there was no clear patient involvement. Some information on rigor of development could be improved to score with the highest standards. Clarity of presentation and applicability also had some room for improvement; however, editorial independence was a concern given the nature of organization, funding, and competing interest issues. This resulted in an overall rating of 5, with recommendation of using it with modifications.

“CANMAT Task Force Report on Cannabis Use”, developed by CANMAT researchers, was focused on people with Bipolar Disorder, Major Depressive Disorder, and Cannabis Use. It scored robustly in methodology and rigor of development. However, information about external reviewing and updating were not provided. It scored moderately in stakeholder involvement and applicability domains, which supported the overall rating of five, with recommendations to implement with modifications.

“SAMHSA Substance Use Disorder Treatment for People with Co-Occurring Disorders” was developed by SAMHSA focusing on people with co-occurring disorders. It focused on a broad target of population; however, it had one of the lowest scoring guidelines across all the domains. Overall rating was three, with recommendation of use with modifications.

“ESTSS Recommendations for PTSD with Comorbid SUD” was focused on people with PTSD and SUD. It scored moderately in scope and purpose and clarity. Otherwise, this guideline had multiple areas in different domains that could be improved to score better. Overall rating was three, with the recommendation to be used after modifications.

“SAMHSA Treatment Considerations for Youth” by SAMHSA focused on youth and young adults and scored high in many domains, including rigor, clarity, and applicability; however, editorial independence scores were very low. Overall rating was five; however, the recommendation is to use with modifications and focus on possible bias due to unclear editorial independence.

“Australian Guidelines on Management of Co-Occurring Conditions” scored the highest overall score of six but still had weak rigor of development and was recommended to use with possible modifications.

### 3.2. Guidelines Not Appraised

There were multiple forms of recommendations, guidance, and information resources related to the management of concurrent disorders that we did not include in this study to appraise by AGREE II, as some of them were not clinical management formal guidelines on concurrent disorder but had partial guidance on elements of concurrent disorder management [34]. While some of them were formal clinical guidelines, their primary scope was not primarily a concurrent disorder, despite having some specific information related to the management of concurrent disorder [35].

As in previous search results, many of the resources had very comprehensive information on concurrent disorder management. SAMHSA had several very comprehensive resources; however, they were either not a formal guideline or had a very specific focus, such as only opioid disorder. “New Australian Guidelines for the Treatment of Alcohol Problems: An Overview of Recommendations” had clear focus and recommendations for alcohol use disorder and had a section on understanding and managing comorbidities for people with alcohol problems: polydrug use and dependence, co-occurring mental disorders, and physical comorbidities; however, as concurrent disorders were not a primary focus, they were not included in our appraisal. “Evidence-based Guidelines for the Pharmacological Treatment of Schizophrenia: Updated recommendations from the British Association for Psychopharmacology” had some information addressing concurrent substance use; however, despite the rigorous guideline development and information, it was not included as concurrent disorder was not a primary focus of this guideline. “Co-occurring Depression and Substance Use Disorders in Young People” also had recommendations focused on young people with concurrent disorders; however, the rigor needed for the standards, which is required for the evidence-based guideline development, was lacking [36].

There were multiple management recommendations that were focused only on one mental health- or one substance use-related issue. However, we included, for appraisal only, “Psychological Treatment of PTSD with Comorbid Substance Use Disorder (SUD): Expert Recommendations of the European Society for Traumatic Stress Studies (ESTSS)” as it had unique expert recommendations for the assessment and psychological treatment of PTSD and comorbid SUD with recommendations [31].

“State of the Science: Treatment of Comorbid Posttraumatic Stress Disorder and Substance Use disorders” similarly had a focus of PTSD only [37]. “International Consensus Statement on Screening, Diagnosis and Treatment of Substance Use Disorder Patients with Comorbid Attention Deficit/Hyperactivity Disorder” had a focus on screening, diagnosis, and treatment of substance use disorders in people with ADHD [34]. “Management of nicotine dependence in patients with psychiatric disorders—recommendations of the Polish Psychiatric Association” had a focus of only nicotine dependence [38]. “Enhancing the Social Network: Multimodal Treatment for Comorbid Borderline Personality Disorder and Alcohol Use Disorder” similarly had a very narrow focus on alcohol use disorder in people with borderline personality disorder [39].

American College of Surgeon Trauma Programs “Best Practices Guidelines Screening and Intervention for Mental Health Disorders and Substance Use and Misuse in The Acute Trauma Patient” had a very narrow focus on acute trauma settings only [40]. The British Columbia Center for Substance Abuse in partnership with the Ministry of Health had updated “Guideline for the Clinical Management of Opioid Use Disorder” with the mention of comorbidity management; however, it was not a focus of guideline [41]. WHO’s “Mental Health Gap Action Program (mhGAP) Guideline for Mental, Neurological and Substance Use Disorders” and update with ten priority conditions provides up-to-date WHO guidance to facilitate delivery of mental, neurological, and substance use-related interventions by health workers in low-income and middle-income countries [34].

“Establishing a System of Care for Severe and Refractory Dual Disorder in the State of Hawaii” had a focus on system and policy recommendations [42]. Most recent updated “Canadian guideline for the clinical management of high-risk drinking and alcohol use disorder” and relevant responses by other authors discussed management of comorbid mental health conditions, including pharmacotherapy of depression [43].

Very specific narrow focus of population was addressed in guidelines such as “Substance use and related disorders among persons exposed to the 9/11 terrorist attacks: Essentials for screening and intervention” and “Management of Mental Health Disorders, Substance Use Disorders, and Suicide in Adults with Spinal Cord Injury: Clinical Practice Guideline for Healthcare Providers” [44,45].

Multiple guidelines focusing on specific mental health, such as NICE guidelines “Depression in adults: treatment and management”, “Finding the Right Setting for the Right Treatment During the Acute Treatment of Individuals with Schizophrenia: A Narrative Review and Clinical Practice Guideline”, “The American Psychiatric Association Practice Guideline for the Treatment of Patients With Schizophrenia”, “The Australian evidence-based clinical practice guideline for attention deficit hyperactivity disorder” and “2023 Guidelines on the Diagnosis and Treatment of Insomnia in Adults—Brazilian Sleep Association”, had some recommendations related to concurrent disorders [46,47,48,49,50].

In addition, guidelines that were addressed to allied healthcare workers or families and were not addressed to physicians were not included. Many different resources, such as handbooks, reviews of the current literature, reviews of recommendations, or focused guidelines with very specific information about concurrent disorders as well, were not considered for inclusion for the appraisal [44,48].

## 4. Discussion

This review update addressed new concurrent disorder guidelines developed after our initial systematic review including guidelines from 2000 to 2020. Several new guidelines have been developed since 2020. We have included eight guidelines for appraisal that met the criteria of being a formal clinical recommendation guideline with the primary focus on concurrent mental health and substance use disorders. Although there are multiple guidance and recommendations developed in the last five years, suggesting an increasing trend or recognition of importance, there is still a significant lack of availability of specific, high-quality concurrent disorder guidelines in different countries. All guidelines struggled to utilize a sufficient evidence base, and at times, did not include systematic evidence searches. Overall, there was a lack of rigor even in the newer guidelines.

An important limitation observed across the reviewed guidelines was the minimal inclusion of patient perspectives during guideline development. The involvement of patients is increasingly recognized as essential for ensuring that clinical recommendations reflect real-world treatment needs and barriers to care. In the context of concurrent disorders, where treatment engagement and long-term adherence are often challenging, incorporating lived-experience perspectives may improve acceptability of clinical recommendations. Future guideline development should therefore prioritize structured patient involvement through the guideline development process, including recommendation formulation and implementation planning.

Given the limited number of eligible guidelines and their heterogeneity in scope, population, and development methodology, a formal stratified quantitative analysis by region or target population was not conducted, as this would risk overinterpretation. Instead, subgroup-level patterns were explored narratively, highlighting consistent strengths and weaknesses across national, governmental, and expert consensus.

As in our initial search, all guidelines were ICD (International Classification of Diseases/DSM (The Diagnostic and Statistical Manual of Mental Disorders))-based. Guidelines also focused on specific combinations of disorders. Newer approaches such as the HiTOP (Hierarchical Taxonomy of Psychopathology) model did not utilize any formulation of concurrent disorders in guidelines. Given the lack of diagnostic recommendation from ICD/DSM, most guidelines struggle with the complexity and diversity of dual disorders.

Sequential, parallel, and integrated models are levels of organization and integration of care. Integrated treatment models are considered to provide the best outcomes and are most cost-effective, while offering one team for providing addiction and mental health services within the same setting. Still, there is a need for more guidance and structure related to the mentioned. Currently, the simple four quadrant model is still one of the few guidances for infrastructure needs.

Transparency regarding editorial independence and competing interests remains an important requirement for guideline development. Several of the reviewed guidelines provide limited information about funding sources, potential conflicts of interest, or the influence of supporting organizations. Greater transparency in these areas is essential to ensure credibility of recommendations and to minimize potential bias. In addition, few guidelines describe mechanisms for monitoring clinical implications. The development of measurable implementation indicators would allow healthcare systems to evaluate adherence and assess their real-world impact on patient outcomes.

## 5. Conclusions

Similar to our previous review, the present update demonstrates that comprehensive, high-quality guidance for the management of concurrent disorders remains limited. While some evidence exists for specific treatment approaches within particular disorder combinations, such as different pharmacological responses in individuals with alcohol dependence and major depressive disorder, or the potential benefits of clozapine in schizophrenia with comorbid substance use, these findings remain fragmented and insufficient to support broad clinical guidance for the management of concurrent disorders as a whole.

Evidence increasingly supports integrated models of care as a promising approach for improving outcomes in individuals with concurrent disorders. However, the implementation of integrated treatment services remains rare across many healthcare systems. Key elements necessary for effective clinical decision-making, including treatment complexity, staging, functional assessment, and treatment matching, are largely absent from current guideline recommendations.

Overall, this systematic review update highlights persistent methodological limitations in the development of concurrent disorder management guidelines. The review revealed a limited evidence base, insufficient rigor of development, and gaps in applicability and stakeholder involvement. Furthermore, current guidelines do not address essential aspects of treatment planning, such as concurrent disorder framework specifics, treatment needs “matching”, and the evaluation of illness severity or treatment staging. Future guideline development would benefit from stronger methodological standards, greater transparency in editorial processes, inclusion of patient perspectives, and consideration of financial resources required for implementation. Further research and guideline development should support integrated care models and incorporate frameworks addressing illness staging and treatment complexity. Strengthening these elements may improve the clinical utility of guidelines and support more effective, evidence-informed management of concurrent disorders.

## Figures and Tables

**Table 1:** Concurrent disorder guidelines included for the appraisal with the AGREE II (Appraisal of Guidelines for Research and Evaluation) tool (last reviewed 18 June 2025).

	Title	Developed by	Country	Year	Population Targeted
1	Management of Schizophrenia and Comorbid Substance Use Disorders: Expert Review and Guidance [26]	University Hospitals and Research Institutes in Spain	Spain	2024	People with schizophrenia and substance use disorders
2	Principles of Care for Young Adults with Co-Occurring Psychiatric and Substance Use Disorders [27]	Boston University, Boston Medical Centre, University of California	USA	2021	People with comorbid mental health and substance use disorders
3	Substance Use Disorder Treatment for People with Co-Occurring Disorder TIP 42 [28]	Substance Abuse and Mental Health Services Administration	USA	2020	People with co-occurring substance use disorders and mental health disorders
4	Canadian Network for Mood and Anxiety Treatments (CANMAT) Task Force Report: A Systematic Review and Recommendations of Cannabis use in Bipolar Disorder and Major Depressive Disorder [29]	Canadian Network for Mood and Anxiety Treatments (CANMAT)	Canada	2023	People with Bipolar Disorder, Major Depressive Disorder and Cannabis Use
5	SAMHSA Substance Use Disorder Treatment for People with Co-Occurring Disorders [30]	Substance Abuse and Mental Health Services Administration	USA	2021	People With Co-Occurring Disorders
6	Psychological Treatment of PTSD with Comorbid Substance Use Disorder (SUD): Expert Recommendations of the European Society for Traumatic Stress Studies (ESTSS) [31]	European Society for Traumatic Stress Studies	Europe	2023	People with PTSD with Comorbid Substance Use Disorder
7	Treatment Considerations for Youth and Young Adults with Serious Emotional Disturbances and Serious Mental Illnesses and Co-occurring Substance Use [32]	Substance Abuse and Mental Health Services Administration	USA	2021	Youth and Young Adults with Serious Emotional Disturbances and Serious Mental Illnesses and Co-occurring Substance Use
8	Guidelines on the Management of Co-Occurring Alcohol and other Drug and Mental Health Conditions in Alcohol and other Drug Treatment Settings [33]	Australian Government	Australia	2022	People with Co-Occurring Alcohol and other Drug and Mental Health Conditions in Alcohol and other Drug Treatment Settings

**Table 2:** Full version of the AGREE II instrument (Strongly Disagree—1, Strongly Agree—7).

GUIDELINES (Please See Table 1: Included Guidelines)	1	2	3	4	5	6	7	8
**DOMAIN 1. SCOPE AND PURPOSE**								
1. The overall objective(s) of the guideline is (are) specifically described.	5	5	6	6	5	3	6	6
2. The health question(s) covered by the guideline is (are) specifically described.	5	6	6	5	5	5	5	6
3. The population (patients, public, etc.) to whom the guideline is meant to apply is specifically described.	2	4	5	5	3	3	6	7
**DOMAIN 2. STAKEHOLDER INVOLVEMENT**								
4. The guideline development group includes individuals from all relevant professional groups.	2	4	7	6	2	4	5	6
5. The views and preferences of the target population (patients, public, etc.) have been sought.	2	1	2	5	2	3	4	6
6. The target users of the guideline are clearly defined.	1	2	7	3	3	2	6	6
**DOMAIN 3. RIGOR OF DEVELOPMENT**								
7. Systematic methods were used to search for evidence.	6	2	2	6	1	3	6	2
8. The criteria for selecting the evidence are clearly described.	4	1	1	6	1	4	6	1
9. The strength and limitations of the body of evidence are clearly described.	1	2	1	7	1	4	5	4
10. The methods for formulating the recommendations are clearly described.	1	1	4	7	1	2	6	5
11. The health benefits, side effects, and risks have been considered in formulating the recommendations.	4	3	6	6	2	3	4	4
12. There is an explicit link between the recommendations and the supporting evidence.	3	6	5	6	4	6	6	6
13. The guideline has been externally reviewed by experts prior to its publication.	1	1	4	1	1	1	1	1
14. A procedure for updating the guideline is provided.	1	1	2	1	1	1	1	2
**DOMAIN 4. CLARITY OF PRESENTATION**								
15. The recommendations are specific and unambiguous.	3	5	6	6	5	6	6	7
16. The different options for management of the condition or health issue are clearly presented.	3	2	5	6	4	4	6	7
17. Key recommendations are easily identifiable.	2	3	6	5	6	4	6	7
**DOMAIN 5. APPLICABILITY**								
18. The guideline describes facilitators and barriers to its application.	2	4	3	6	5	3	6	6
19. The guideline provides advice and/or tools on how the recommendations can be put into practice.	4	5	6	6	4	2	5	6
20. The potential resource implications of applying the recommendations have been considered.	2	4	3	2	4	2	6	5
21. The guideline presents monitoring and/or auditing criteria.	1	1	2	1	1	1	5	5
**DOMAIN 6. EDITORIAL INDEPENDENCE**								
22. The views of the funding body have not influenced the content of the guideline.	2	5	1	3	2	1	2	4
23. Competing interests of guideline development group members have been recorded and addressed.	4	6	1	4	1	3	2	1
1. Rate the overall quality of this guideline. Lowest possible quality—1 Highest possible quality—7	3	4	5	5	3	3	5	6
OVERALL CALCULATED BY DOMAIN AVERAGE								
2. I would recommend this guideline for use. Yes—1, Yes with Modifications—2, No—3	2	2	2	2	2	2	2	2
NOTES								

**Table 3:** Short version of the Agree II instrument (Strongly Disagree—1, Strongly Agree—7).

GUIDELINES (Please See Table 1: Included Guidelines)	1	2	3	4	5	6	7	8
**DOMAIN 1. SCOPE AND PURPOSE**	4	5	6	5	4	4	6	6
**DOMAIN 2. STAKEHOLDER INVOLVEMENT**	2	2	5	5	2	3	5	6
**DOMAIN 3. RIGOR OF DEVELOPMENT**	3	2	3	6	2	3	6	4
**DOMAIN 4. CLARITY OF PRESENTATION**	3	3	6	6	5	5	6	7
**DOMAIN 5. APPLICABILITY**	2	4	4	4	4	2	6	6
**DOMAIN 6. EDITORIAL INDEPENDENCE**	3	6	1	4	2	2	2	3
1. Rate the overall quality of this guideline. Lowest possible quality—1 Highest possible quality—7	3	4	5	5	3	3	5	6
2. I would recommend this guideline for use. Yes—1, Yes with Modifications—2, No—3	2	2	2	2	2	2	2	2
NOTES								

## Data Availability

All relevant data are included in this article and Supplementary Information Files. Additional data are available upon request.

## Supplementary Materials

The following supporting information can be downloaded at https://www.mdpi.com/article/10.3390/jcm15083123/s1, PRISMA 2020 Checklist.
