# Supplementary material for: Concurrent (Dual) Disorder Management Guidelines: Systematic Review Update
Source: J Clin Med. 2026 Apr 20;15(8):3123. doi: 10.3390/jcm15083123 (PMC13117480; doi:10.3390/jcm15083123)
Supplement: Supplementary file 1 [file jcm-15-03123-s001.zip › jcm-4083562-supplementary.pdf]

# PRISMA 2020 Checklist

| Section and Topic | Item # | Checklist item | Location where item is reported |
|---|---|---|---|
| **TITLE** | | | |
| Title | 1 | Identify the report as a systematic review.<br><br>**Concurrent (Dual) Disorder Management Guidelines: Systematic Review Update.** | Title |
| **ABSTRACT** | | | |
| Abstract | 2 | See the PRISMA 2020 for Abstracts checklist.<br><br>**Background/Objectives:** The initial systematic review of "Concurrent Disorder Management Guidelines. Systematic Review" assessed the quality of the concurrent disorders' clinical management guidelines in 2020, including the guidelines in the field from 2000-2020. Twenty-four guidelines were identified and assessed with the AGREE II (Appraisal of Guidelines for Research and Evaluation). As dual disorder needs increased specifically among the younger population, requiring significant healthcare resources, more efficient approaches targeting complex concurrent disorders are essential. Since 2020, multiple new guidelines were developed in response to new developments in the field of substance use disorder management. This systematic review update aimed to identify and appraise all new available concurrent disorder management guidelines to strategize the management of concurrent disorders, support better outcomes and further research directions. **Methods:** The review was registered and protocol is available in the international register—PROSPERO (http://www.crd.york.ac.uk/prospero; ID: CRD420251076197). Literature searches were performed by two independent authors in electronic databases, web, and gray literature. The inclusion criteria were English language clinical management guidelines for adult concurrent disorders between 2020-2025. Sources, that were not formal clinical guidelines and were not addressed to physicians for adult age group, were addressed to intellectual/developmental disability, and were not in English, were excluded. **Results:** The initial search resulted in 5003 records. A total of eight new guidelines were identified and assessed with the AGREE II, highlighting the consistent gap in the evidence-based management recommendations. **Conclusions:** Guidelines had similar to 2020 findings supporting dual or combined treatment, however all guidelines had multiple domains not developed rigorously and with methodological limitations. Levels of complexity and staging of treatment were not considered in recommendations. Average domain scores were very low, with the lowest being applicability and editorial independence. Development of high quality, rigorously developed, evidence-based guidelines, also addressing staging, resource implications, and patient involvement is recommended as the evidence base remains underdeveloped. **Funding:** This research received the following fundings: 1. S.H. as a Ph.D. student received previously a Four-Year Fellowship from The University of British Columbia Graduate and Postdoctoral Studies; 2. By World federation of Societies of Biological psychiatry (WFSBP) and an unrestricted fund from Richter Gedeon. | Abstract |
| **INTRODUCTION** | | | |
| Rationale | 3 | Describe the rationale for the review in the context of existing knowledge.<br><br>An updated synthesis of new management of concurrent disorder is critical to inform the practice to address the increasing burden due to increasing number of people affected by concurrent disorder and rapid changes in the field. | Introduction |
| Objectives | 4 | Provide an explicit statement of the objective(s) or question(s) the review addresses.<br><br>This systematic review aims to update and systematically evaluate, appraise new published concurrent disorder clinical management guidelines after March 18th, 2020, since the first published systematic review, addressing the management of concurrent disorders in adult population. The goal was to follow the original systematic review protocol and focus on concurrent disorder guidelines in English language for healthcare professionals in primary, secondary, tertiary care, but not to patients and their families, guidelines for persons with neurodevelopmental disorders and/or intellectual/developmental disabilities occurring simultaneously with concurrent disorder. The update objective is to explore guidelines' overall scope, approach, structure, knowledge limitations, consistency, methodological issues, potential bias issues, or other potential issues. | Introduction |

PRISMA 2020 Checklist

| Section and Topic | Item # | Checklist item | Location where item is reported |
|---|---|---|---|
| **METHODS** | | | |
| Eligibility criteria | 5 | Specify the inclusion and exclusion criteria for the review and how studies were grouped for the syntheses.<br><br>The inclusion criteria included all published and unpublished English language clinical management guidelines of concurrent disorders, that were published by professional organizations (e.g., American Psychiatric Association, World Health Organisation, etc.), were developed through systematic evidence review, expert consensus, formal approval process, and serve as authoritative standards of care.<br>We included concurrent disorders' formal clinical management guidelines for the appraisal of guidelines with the AGREE II (Appraisal of Guidelines for REsearch and Evaluation) tool. The AGREE (Appraisal of Guidelines for REsearch and Evaluation) instrument was developed to address the issue of variability in guideline quality, which assesses the methodological rigor and transparency of guideline development. The original AGREE tool has been refined to AGREE II.<br>We did not include sources from UpToDate or DynaMed for the appraisal as the resources there are not considered to be formal guidelines, while they are evidence-based, continuously updated clinical reference tools. Multiple guidelines had some information about management of concurrent disorders, however, that was not their primary focus, and so they were not included for the appraisal by AGREE II. Similarly, the guidelines that were addressed to patients and their families, all relevant professionals, were considered for the review, while they were not appraised with the AGREE II. For the purposes of this review, similar to the original review, developmental/intellectual disabilities occurring simultaneously with mental health concerns, confusingly also labelled "dual diagnosis" or "concurrent disorder", were not considered. Accordingly, the exclusion criteria were: general reviews of concurrent disorder management, literature that was not a formal guideline (although some of the guidelines were not classic formal guideline and had focused review of available evidence as evidence base for the recommendations) non-English guidelines, literature without concurrent disorders as their primary focus, literature addressing persons with intellectual/developmental disabilities and concurrent mental disorders, and literature that was published prior to 18 March 2020. | Methods |
| Information sources | 6 | Specify all databases, registers, websites, organisations, reference lists and other sources searched or consulted to identify studies. Specify the date when each source was last searched or consulted.<br><br>The following electronic databases and websites were used to extract guidelines: MEDLINE (via Ovid), EMBASE (via Ovid), CINAHL, PsycINFO, JouleCMA, Trip, DynaMed, NICE Guidelines, SIGN, CADTH, and UpToDate. Additionally, a web search for relevant reference lists and other gray literature was done by all authors. Clinicians and researchers in the field were contacted as well in search of known information about the guidelines in the field. All the searches were set between 18 March 2020 until 18 June 2025. | Methods |
| Search strategy | 7 | Present the full search strategies for all databases, registers and websites, including any filters and limits used.<br><br>To identify novel guidelines in the field, two independent reviewers: S.H. and Z.A completed literature searches and subsequent appraisals. When there was a disagreement, S.L.C. was involved, and, if any discrepancy, C.G.S. advised for a final decision.<br><br>**Supplementary Materials:** The following supporting information can be downloaded at: https://www.mdpi.com/article/doi/s1<br><br>EMBASE/Ovid Search Terms Used and Results; MEDLINE/Ovid Search Terms Used and Results; PsychINFO Search Terms Used and Results and CINAHL Search Terms Used and Results.<br>**Appendix A.1**<br>**Covidence Search Terms:**<br><br>("mental disorder*" OR "psychiatric disorder*" OR "mental illness*" OR "psychological disorder*" OR "mood disorder*" OR depression OR "bipolar disorder*" OR mania OR "anxiety disorder*" OR PTSD OR "post-traumatic stress disorder*" OR "schizophrenia" OR schizo* OR psychosis OR psychotic OR "personality disorder*" OR "eating disorder*" OR anorexia OR bulimia OR "sleep disorder*" OR insomnia OR "impulse control disorder*" OR "dissociative disorder*" OR "sexual dysfunction*" OR "somatoform disorder*" OR "stress-related disorder*" OR trauma) AND ("substance use disorder*" OR "substance abuse" OR "drug abuse" OR "alcohol use disorder*" OR alcoholism OR "tobacco use disorder*" OR smoking OR nicotine OR cannabis OR marijuana OR heroin OR cocaine OR methamphetamine OR "crystal meth" OR stimulant* | Methods |

# PRISMA 2020 Checklist

| Section and Topic | Item # | Checklist item | Location where item is reported |
|---|---|---|---|
| | | OR depressant* OR hallucinogen* OR LSD OR MDMA OR opioids OR opiate* OR narcotic* OR "inhalant abuse" OR "prescription drug misuse") AND ("concurrent disorder*" OR "co-occurring disorder*" OR "dual diagnosis" OR "comorbid*" OR "co-existing disorder*" OR "co-presenting disorder*" OR "substance-induced mental disorder*") AND (guideline* OR "practice guideline*" OR "best practice*" OR "clinical recommendation*" OR consensus OR "management guideline*" OR management).<br><br>**Manual search of databases not allowing Boolean**<br><br>Keywords: concurrent disorder; co-occurring disorder; dual diagnosis; dual pathology; addiction comorbidity; comorbid substance abuse; comorbid illicit use; comorbid addiction; comorbid mental illness; coexisting mental illness.<br>Inclusion and Exclusion criteria as per "Guideline Update – Exclusion Hierarchy"<br>Search dates: August 1 – 7, 2025 inclusive<br><br>**Trip**<br>1. Screening and intervention for mental health disorders and substance use and misuse in the acute trauma patient, 2022. American College of Surgeons<br>2. Recommendations on Increasing Access to Mental Health and Substance Use Disorder Treatment Facilities and Programs for Persons with Bleeding Disorders, 2025. National Hemophilia Foundation<br>3. Mental Health Gap Action Programme (mhGAP) guideline for mental, neurological and substance use disorders, 2023. World Health Organisation Guidelines<br>4. Substance abuse treatment for persons with co-occurring disorders, 2005. Substance Abuse and Mental Health Services Administration (U.S.)<br>5. Medication-assisted treatment for opioid addiction in opioid treatment programs: Treatment of co-occurring disorders, 2005. Substance Abuse and Mental Health Services Administration (U.S.)<br>**JouleCMA**<br>None<br><br>**SIGN**<br>None<br><br>**UpToDate**<br>1. Co-occurring substance use and posttraumatic stress disorder in adults<br><br>2. Co-occurring schizophrenia and substance use disorder: Epidemiology, clinical features, assessment, and diagnosis<br>https://www.uptodate.com/contents/co-occurring-schizophrenia-and-substance-use-disorder-epidemiology-clinical-features-assessment-and-diagnosis?search=co-occurring%20disorder&source=search_result&selectedTitle=3~140&usage_type=default&display_rank=3<br>3. Co-occurring schizophrenia and substance use disorder: Pharmacotherapy<br>https://www.uptodate.com/contents/co-occurring-schizophrenia-and-substance-use-disorder-pharmacotherapy?search=co-occurring%20disorder&source=search_result&selectedTitle=6~140&usage_type=default&display_rank=6<br>4. Co-occurring schizophrenia and substance use disorder: Psychosocial interventions<br>https://www.uptodate.com/contents/co-occurring-schizophrenia-and-substance-use-disorder-psychosocial-interventions?search=co-occurring%20disorder&source=search_result&selectedTitle=5~140&usage_type=default&display_rank=5<br><br>**NICE Guidelines** | |

| Section and Topic | Item # | Checklist item | Location where item is reported |
|---|---|---|---|
| | | 1. Coexisting severe mental illness and substance misuse: community health and social care services, Published November 30, 2016. A presentational change was made on August 14, 2024. There were no changes to practice.<br>**CADTH**<br>None<br><br>**Dynamed**<br>1. Co-occurring Substance Use Disorder and Mental Health Disorder" https://www.dynamed.com/condition/co-occurring-substance-use-disorder-and-mental-health-disorder#GUID-D56BCA86-A0FB-45CC-86BC-1F5ADA4E700E<br><br>The focus was to find guidelines with set inclusion criteria, which included all published and unpublished English language clinical management guidelines of concurrent disorders, that were published by professional organizations (e.g., American Psychiatric Association, World Health Organisation, etc.), were developed through systematic evidence review, expert consensus, formal approval process, and serve as authoritative standards of care. All the searches were set between 18 March 2020 until 18 June 2025. | |
| Selection process | 8 | Specify the methods used to decide whether a study met the inclusion criteria of the review, including how many reviewers screened each record and each report retrieved, whether they worked independently, and if applicable, details of automation tools used in the process.<br><br>The search with the above criteria conducted revealed a total of 5003 results, comprising 4572 from an electronic database search and 431 from a grey literature search. The 4572 results from the electronic database search were all imported to Covidence. Covidence automatically identified 1041 duplicates, 19 duplicates were identified manually, all of which were deleted. As a result, only 3512 results remained in Covidence. The results of the grey literature and website search from different sources, including a checking for updates on the appraised 24 papers from the original review (in total 431 results) were not uploaded to Covidence. Whenever possible, the removal of duplicate results in the grey literature search was done manually and assessed with the same approach. From both sources, the electronic database and grey literature/website searches, the study titles, abstracts, and full papers were examined by two reviewers (S.H. and Z.A.) to identify eligible studies based on the described inclusion criteria. Decisions of the two authors were recorded separately and in case of disagreement, were discussed. In the absence of consensus, a decision was made by the third reviewer (S.L.C.), and finally, by the supervisory author (C.G.S.). All titles were scanned (3943) and if relevant to concurrent disorders, abstracts were read. Studies were classified for inclusion to appraise into YES (13), MAYBE (88) and NO (3842) groups. Electronic database search results were manually sorted within Covidence, while grey literature and website results were manually sorted outside of it. In the YES and MAYBE groups, 100 full papers (80 from an electronic database + 20 from gray literature and websites***) were read. A full-text review was performed for the 100 selected studies and recorded in Covidence or into a study selection form, documenting the reason for the exclusion and inclusion of each study. After this process, 13 papers remained that fulfilled inclusion criteria and were considered for the qualitative analysis. After full assessment, only eight papers fully fulfilled the inclusion criteria and were included in the final appraisal (Figure 1: PRISMA Flow Diagram 1, Table 1). The AGREE II instrument was used to report the guidelines. | Methods |
| Data collection process | 9 | Specify the methods used to collect data from reports, including how many reviewers collected data from each report, whether they worked independently, any processes for obtaining or confirming data from study investigators, and if applicable, details of automation tools used in the process.<br><br>Two independent reviewers: S.H. and Z.A after completed literature searches, collected data separately from studies and completed subsequent appraisals individually. Later they met and discussed all their findings. When there was a disagreement, S.L.C., third reviewer was involved, and, if any discrepancy, C.G.S. advised for a final decision.<br>Reviewers worked independently creating separate data forms. For each study appraisal both reviewers created individual study appraisal forms. | Methods |

| Section and Topic | Item # | Checklist item | Location where item is reported |
|---|---|---|---|
| | | After meeting, discussion and agreement, combined data for AGREE II tables were created. | |
| Data items | 10a | List and define all outcomes for which data were sought. Specify whether all results that were compatible with each outcome domain in each study were sought (e.g. for all measures, time points, analyses), and if not, the methods used to decide which results to collect.<br><br>AGREE II Domains were assessed (Short version of Domains as below):<br>1. Scope and Purpose<br>2. Stakeholder Involvement<br>3. Rigor of Development<br>4. Clarity of Presentation<br>5. Applicability<br>6. Editorial Independence<br>The long detailed version of AGREE II Domain outcomes are presented in Table 2 Full version of the AGREE II instrument (Strongly Disagree—1, Strongly Agree—7).<br>Every single Domain subheading appraisal result was discussed and agreed between two reviewers (S.H. and Z.A.), and if disagreement, third reviewer (S.L.C.), and eventually supervisor (C.G.S.) was involved. | Methods |
| | 10b | List and define all other variables for which data were sought (e.g. participant and intervention characteristics, funding sources). Describe any assumptions made about any missing or unclear information.<br><br>Any missing or unclear information was evaluated according to the AGREE II recommendations. | Methods |
| Study risk of bias assessment | 11 | Specify the methods used to assess risk of bias in the included studies, including details of the tool(s) used, how many reviewers assessed each study and whether they worked independently, and if applicable, details of automation tools used in the process.<br><br>Two independent reviewers: S.H. and Z.A after completed literature searches, collected data separately from studies and completed subsequent appraisals individually. Later they met and discussed all their findings. When there was a disagreement, S.L.C., third reviewer was involved, and, if any discrepancy, C.G.S. advised for a final decision.<br>AGREE II was uses to appraise all guidelines by two reviewers independently. Reviewers worked independently creating separate data forms. For each study appraisal both reviewers created individual study appraisal forms. After meeting, discussion and agreement, combined data for AGREE II tables were created. | Methods |
| Effect measures | 12 | Specify for each outcome the effect measure(s) (e.g. risk ratio, mean difference) used in the synthesis or presentation of results.<br><br>N/A. | Methods |
| Synthesis methods | 13a | Describe the processes used to decide which studies were eligible for each synthesis (e.g. tabulating the study intervention characteristics and comparing against the planned groups for each synthesis (item #5)).<br><br>N/A | Methods |
| | 13b | Describe any methods required to prepare the data for presentation or synthesis, such as handling of missing summary statistics, or data conversions. | Methods |

# PRISMA 2020 Checklist

| Section and Topic | Item # | Checklist item | Location where item is reported |
|---|---|---|---|
| | | N/A | |
| | 13c | Describe any methods used to tabulate or visually display results of individual studies and syntheses.<br><br>PRISMA Flow Diagram was used to report study numbers.<br>Tables were used to report some study characteristics and AGREE II Domain details. | Methods |
| | 13d | Describe any methods used to synthesize results and provide a rationale for the choice(s). If meta-analysis was performed, describe the model(s), method(s) to identify the presence and extent of statistical heterogeneity, and software package(s) used.<br><br>PRISMA Flow Diagram was used to report study numbers, facilitated through Covidence due to clarify of presenting all studies in an organized fashion.<br>Tables were used to report some study characteristics and AGREE II Domain details. Initially each reviewer created separate results for each AGREE II domain details as per AGREE II guide. After discussion of two results, final decision was made based on two results. | Methods |
| | 13e | Describe any methods used to explore possible causes of heterogeneity among study results (e.g. subgroup analysis, meta-regression).<br><br>N/A. | Methods |
| | 13f | Describe any sensitivity analyses conducted to assess robustness of the synthesized results.<br><br>N/A. | Methods |
| Reporting bias assessment | 14 | Describe any methods used to assess risk of bias due to missing results in a synthesis (arising from reporting biases).<br><br>N/A. | Methods |
| Certainty assessment | 15 | Describe any methods used to assess certainty (or confidence) in the body of evidence for an outcome.<br><br>N/A. | Methods |
| **RESULTS** | | | |
| Study selection | 16a | Describe the results of the search and selection process, from the number of records identified in the search to the number of studies included in the review, ideally using a flow diagram | Results |

| Section and Topic | Item # | Checklist item | Location where item is reported |
|---|---|---|---|
| | | 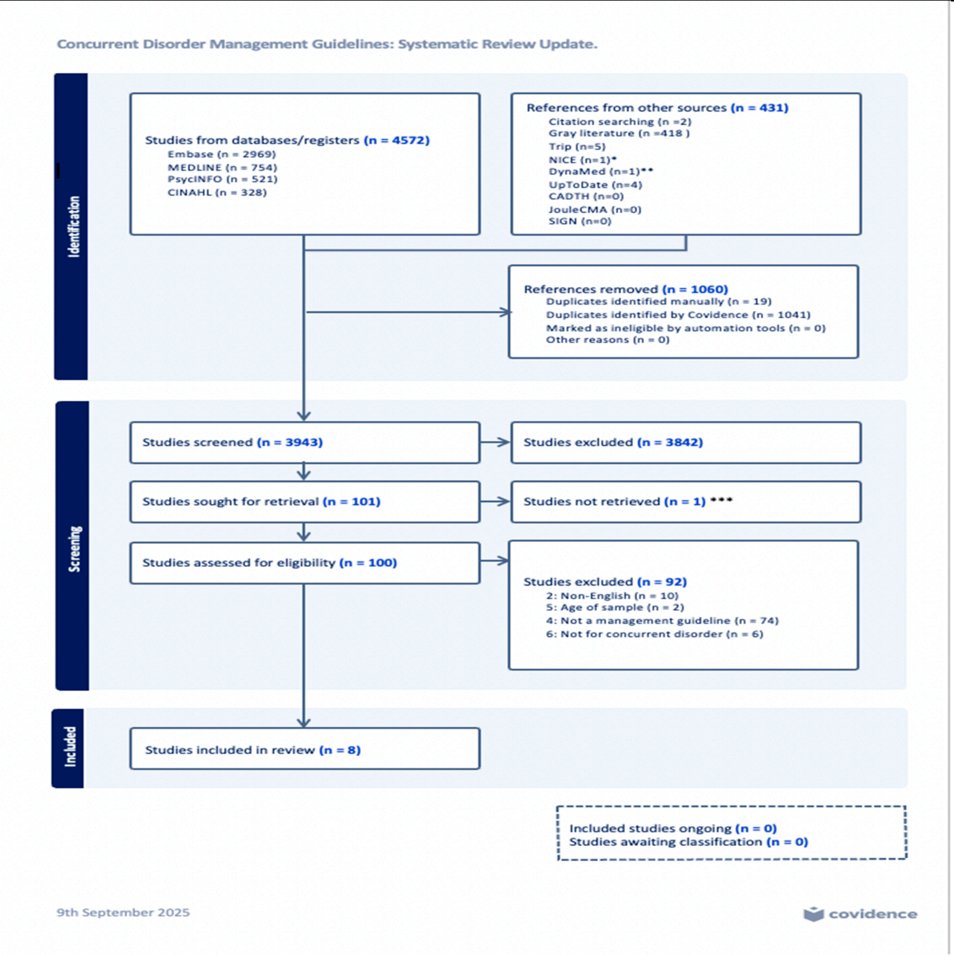 <br>**Figure 1.** PRISMA Flow Diagram: retrieved from Covidence.<br>1. * Coexisting severe mental illness and substance misuse: community health and social care services, Published November 30, 2016 (A presentational change was made on August 14, 2024. There were no changes to practice).<br>2. ** “Co-occurring Substance Use Disorder and Mental Health Disorder” https://www.dynamed.com/condition/cAo-occurring-substance-use-disorder-and-mental-health-disorder<br>3. *** Not available. | |

# PRISMA 2020 Checklist

| Section and Topic | Item # | Checklist item | Location where item is reported |
|---|---|---|---|
| | 16b | Cite studies that might appear to meet the inclusion criteria, but which were excluded, and explain why they were excluded.<br><br>There were multiple forms of recommendations, guidance, and information resources related to the management of concurrent disorders, however we did not include in this study to appraise by AGREE II, as some of them were not clinical management formal guidelines on concurrent disorder but had a partial guidance on elements of concurrent disorder management. While some of them were formal clinical guidelines, their primary scope was not primarily a concurrent disorder, despite they had some specific information related to the management of concurrent disorder. As in previous search results, this time as well, many of the resources had very comprehensive information on concurrent disorder management. SAMHSA had several very comprehensive resources; however, they were either not a formal guideline or had a very specific focus, such as only opioid disorder. “New Australian Guidelines for the Treatment of Alcohol Problems: An Overview of Recommendations” had clear focus and recommendations for alcohol use disorder and had a section on understanding and managing comorbidities for people with alcohol problems: polydrug use and dependence, co-occurring mental disorders, and physical comorbidities, however as concurrent disorders were not a primary focus, it was not included in our appraisal. “Evidence-based Guidelines for the Pharmacological Treatment of Schizophrenia: Updated recommendations from the British Association for Psychopharmacology” had some information addressing concurrent substance use, however despite the rigorous guideline development and information, it was not included as concurrent disorder was not a primary focus of this guideline.<br><br>“Co-occurring Depression and Substance Use Disorders in Young People” also had recommendations focused on young people with concurrent disorders, however the rigor needed for the standards, that is required for the evidence-based guideline development, was lacking [17].<br><br>There were multiple management recommendations that were focused only on one mental health or one substance use related issues. However, we included for appraisal only “Psychological Treatment of PTSD with Comorbid Substance Use Disorder (SUD): Expert Recommendations of the European Society for Traumatic Stress Studies (ESTSS)” as it had unique expert recommendations for the assessment and psychological treatment of PTSD and comorbid SUD with recommendations [18]. “State of the Science: Treatment of Comorbid Posttraumatic Stress Disorder and Substance Use disorders”, similarly had a focus of PTSD only [19].<br><br>“International Consensus Statement on Screening, Diagnosis and Treatment of Substance Use Disorder Patients with Comorbid Attention Deficit/Hyperactivity Disorder” had a focus on screening, diagnosis, and treatment of substance use disorders in people with ADHD [20].<br><br>“Management of nicotine dependence in patients with psychiatric disorders – recommendations of the Polish Psychiatric Association” had a focus of only nicotine dependence [21].<br><br>“Enhancing the Social Network: Multimodal Treatment for Comorbid Borderline Personality Disorder and Alcohol Use Disorder” similarly had a very narrow focus on alcohol use disorder in people with borderline personality disorder [22].<br><br>American College of Surgeon Trauma Programs “Best Practices Guidelines Screening and Intervention for Mental Health Disorders and Substance Use and Misuse in The Acute Trauma Patient” had a very narrow focus on acute trauma settings only [23].<br><br>British Columbia Centre for Substance Abuse in partnership with Ministry of Health had updated “Guideline for the Clinical Management of Opioid Use Disorder” with the mention of comorbidity management, however, it was not a focus of guideline [24].<br><br>WHO’s “Mental Health Gap Action Program (mhGAP) Guideline for Mental, Neurological and Substance Use Disorders”, and update with ten priority conditions provides up-to-date WHO guidance to facilitate delivery of mental, neurological, and substance use related interventions by health workers in low-income and middle-income countries [25].<br><br>“Establishing a System of Care for Severe and Refractory Dual Disorder in the State of Hawaii” had a focus on system and policy recommendations [26]. | Results, References |

| Section and Topic | Item # | Checklist item | Location where item is reported |
|---|---|---|---|
| | | Most recent updated “Canadian guideline for the clinical management of high-risk drinking and alcohol use disorder” and relevant responses by other authors discussed about management of comorbid mental health conditions, including pharmacotherapy of depression [27].<br><br>Very specific narrow focus of population was addressed in guidelines such as “Substance use and related disorders among persons exposed to the 9/11 terrorist attacks: Essentials for screening and intervention” and “Management of Mental Health Disorders, Substance Use Disorders, and Suicide in Adults with Spinal Cord Injury: Clinical Practice Guideline for Healthcare Providers” [28, 29].<br><br>Multiple guidelines focusing on specific mental health, such as NICE guidelines “Depression in adults: treatment and management”, “Finding the Right Setting for the Right Treatment During the Acute Treatment of Individuals with Schizophrenia: A Narrative Review and Clinical Practice Guideline”, “The American Psychiatric Association Practice Guideline for the Treatment of Patients With Schizophrenia”, “The Australian evidence-based clinical practice guideline for attention deficit hyperactivity disorder”, “2023 Guidelines on the Diagnosis and Treatment of Insomnia in Adults – Brazilian Sleep Association” had some recommendations related to concurrent disorders [30-34].<br><br>In addition, guidelines that were addressed to allied healthcare workers or families and were not addressed to physicians, were not included. Many different resources, such as handbooks, reviews of current literature, reviews of recommendations, or focused guidelines with very specific some information about concurrent disorders as well were not considered for inclusion for the appraisal [35-36].<br><br>17. Dreyer-Oren, S. E., Amer, E. E., Abrantes, A. M. 2024. Co-occurring depression and substance use disorders in young people. Psychiatric Annals, 54(9), e253- e257. https://doi.org/10.3928/00485713-20240913-03.<br>18. Roberts NP, Lotzin A, Schäfer I. Psychological treatment of PTSD with comorbid substance use disorder (SUD): expert recommendations of the European Society for Traumatic Stress Studies (ESTSS). Eur J Psychotraumatol. 2023;14(2):2265773. doi: 10.1080/20008066.2023.2265773. Epub 2023 Oct 13. PMID: 37830207; PMCID: PMC10578096.<br>19. Back SE, Jarnecke AM, Norman SB, Zaur AJ, Hien DA. State of the Science: Treatment of comorbid posttraumatic stress disorder and substance use disorders. J Trauma Stress. 2024 Dec;37(6):803-813. doi: 10.1002/jts.23049. Epub 2024 Jun 10. PMID: 38857125.<br>20. Crunelle CL, van den Brink W, Moggi F, Konstenius M, Franck J, Levin FR, van de Glind G, Demetrovics Z, Coetzee C, Luderer M, Schellekens A; ICASA consensus group; Matthys F. International Consensus Statement on Screening, Diagnosis and Treatment of Substance Use Disorder Patients with Comorbid Attention Deficit/Hyperactivity Disorder. Eur Addict Res. 2018;24(1):43-51. doi: 10.1159/000487767. Epub. 2018 Mar 6. PMID: 29510390; PMCID: PMC5986068.<br>21. Wojnar M, Wierzbiński P, Samochowiec J, Rymaszewska J, Filipiak KJ, Wichniak A, Mróz R, Mamcarz A, Dudek D. Management of nicotine dependence in patients with psychiatric disorders - recommendations of the Polish Psychiatric Association - part I. Psychiatr. 2024 Jun 30;58(3):403-418. English, Polish. doi: 10.12740/PP/OnlineFirst/161773. PMID: 37370218.<br>22. Patzelt EH, Conway S, Mermin SA, Jurist J, Choi-Kain LW. Enhancing the Social Network: Multimodal Treatment for Comorbid Borderline Personality Disorder and Alcohol Use Disorder. Am J Psychother. 2025 Mar 1;78(1):55-62. doi: 10.1176/appi.psychotherapy.20230046. Epub 2025 Feb 4. PMID: 39901760.<br>23. American College of Surgeon Trauma Programs “Best Practices Guidelines Screening and Intervention for Mental Health Disorders and Substance Use and Misuse in The Acute Trauma Patient. https://www.facs.org/media/nrcj31ku/mental-health-guidelines.pdf.<br>24. British Columbia Centre for Substance Abuse in partnership with British Columbia Ministry of Health Guideline for the Clinical Management of Opioid Use Disorder. 2023.<br>25. World Health Organization. Mental Health Gap Action Program (mhGAP) Guideline for Mental, Neurological and Substance Use Disorders. ISBN: 9789240084278. 2023.<br>26. Busch G, Seo JY. Establishing a System of Care for Severe and Refractory Dual Disorder in the State of Hawai'i. Hawaii. J Health Soc Welf. 2022 Dec;81(12 Suppl 3):19-26. PMID: 36660278; PMCID: PMC9783815.<br>27. Wood E, Bright J, Hsu K, Goel N, Ross JWG, Hanson A, Teed R, Poulin G, Denning B, Corace K, Chase C, Halpape K, Lim R, Kealey T, Rehm J; Canadian Alcohol Use Disorder Guideline Committee. Canadian guideline for the clinical management of high-risk drinking and alcohol use disorder. CMAJ. 2023 Oct 16;195(40):E1364-E1379. doi: 10.1503/cmaj.230715. PMID: 37844924; PMCID: PMC10581718. | |

| Section and Topic | Item # | Checklist item | Location where item is reported |
|---|---|---|---|
| | | 28. Dowling FG; Lowe SM. Substance Use and Related Disorders Among Persons Exposed to the 9/11 Terrorist Attacks: Essentials for Screening and Intervention. 2023/05/28. Pages in Document: 261-266. https://stacks.cdc.gov/view/cdc/207861.<br>29. Bombardier CH, Azuero CB, Fann JR, Kautz DD, Richards JS, Sabharwal S. Management of Mental Health Disorders, Substance Use Disorders, and Suicide in Adults with Spinal Cord Injury: Clinical Practice Guideline for Healthcare Providers. Top Spinal Cord Inj Rehabil. 2021 Spring;27(2):152-224. doi: 10.46292/sci2702-152. PMID: 34108836; PMCID: PMC8152173.<br>30. NICE guidelines "Depression in adults: treatment and management. 2022. Last reviewed: 19 September 2024. https://www.nice.org.uk/guidance/ng222.<br>31. Correll CU, Arango C, Fagiolini A, Giordano GM, Leucht S, Salazar de Pablo G. Finding the Right Setting for the Right Treatment During the Acute Treatment of Individuals with Schizophrenia: A Narrative Review and Clinical Practice Guideline. Neuropsychiatr Dis Treat. 2024 Jun 19;20:1293-1307. doi: 10.2147/NDT.S459450. PMID: 38911102; PMCID: PMC11194005.<br>32. Keepers GA, Fochtmann LJ, Anzia JM, Benjamin S, Lyness JM, Mojtabai R, Servis M, Walaszek A, Buckley P, Lenzenweger MF, Young AS, Degenhardt A, Hong SH; (Systematic Review). The American Psychiatric Association Practice Guideline for the Treatment of Patients With Schizophrenia. Am J Psychiatry. 2020 Sep 1;177(9):868-872. doi: 10.1176/appi.ajp.2020.177901. PMID: 32867516.<br>33. May T, Birch E, Chaves K, Cranswick N, Culnane E, Delaney J, Derrick M, Eapen V, Edlington C, Efron D, Ewais T, Garner I, Gathercole M, Jagadheesan K, Jobson L, Kramer J, Mack M, Misso M, Murrup-Stewart C, Savage E, Sciberras E, Singh B, Testa R, Vale L, Weirman A, Petch E, Williams K, Bellgrove M. The Australian evidence-based clinical practice guideline for attention deficit hyperactivity disorder. Aust N Z J Psychiatry. 2023 Aug;57(8):1101-1116. doi: 10.1177/00048674231166329. Epub 2023 May 30. PMID: 37254562; PMCID: PMC10363932.<br>34. Drager LF, Assis M, Bacelar AFR, Poyares DLR, Conway SG, Pires GN, de Azevedo AP, Carissimi A, Eckeli AL, Pentagna Á, Almeida CMO, Franco CMR, Sobreira EST, Stelzer FG, Mendes GM, Minhoto GR, Linares IMP, Sousa KMM, Gitaí LLG, Sukys-Claudino L, Sobreira-Neto MA, Zanini MA, Margis R, Martinez SCG. 2023 Guidelines on the Diagnosis and Treatment of Insomnia in Adults - Brazilian Sleep Association. Sleep Sci. 2023 Nov 22;16(Suppl 2):507-549. doi: 10.1055/s-0043-1776281. PMID: 38370879; PMCID: PMC10869237.<br>35. The American Psychiatric Association Practice Guideline for the Treatment of Patients with Schizophrenia. George A. Keepers, Laura J. Fochtmann, Joan M. Anzia, Sheldon Benjamin, Jeffrey M. Lyness, Ramin Mojtabai, Mark Servis, (Systematic Review) 2020. Publication: American Journal of Psychiatry. Volume 177, Number 9 https://doi.org/10.1176/appi.ajp.2020.17790.<br>36. SAMHSA Clinical Issues in Intensive Outpatient Treatment for Substance Use Disorders. Brief Report. Substance use and related disorders among persons exposed to the 9/11 terrorist attacks: Essentials for screening and intervention Pages 261-266. 27 Feb 2023. | |
| Study characteristics | 17 | Cite each included study and present its characteristics.<br><br>"Management of Schizophrenia and Comorbid Substance Use Disorders: Expert Review and Guidance" was developed by Spanish researchers from University Hospitals and Research Institutes. The objectives and health questions covered by the guideline were described well, it was targeted for people with schizophrenia and substance use disorders, however the specifics of population were not described in detail. It did not describe stakeholder involvement, scored low on stakeholder involvement, rigor of development, and clear recommendations of who can use guideline was also missing. Despite very comprehensive search, otherwise in the domain development it was not a rigorously developed guideline. The information that presented, was not very clear for clinical practice. The implication for the resources of In order apply the recommendations, considering the resources, was also limited. Lastly, the guideline did not have sufficient information on editorial independence. Overall, the quality was low, recommending use only with major modifications.<br><br>"Principles of Care for Young Adults with Co-Occurring Psychiatric and Substance Use Disorders" which was published by the researchers from Boston University, Boston Medical Centre, and University of California, was targeted on people with comorbid substance use and mental health disorders. The overall objectives, health question, and population to apply a guideline were well described. Although different groups were involved in development, patient preferences and information on the target users of guideline were not described. Recommendations were clearly connected to evidence, however otherwise, the rigor of development other components had low scores in AGREE II standards. On domains, presentations, applicability, and editorial independence, the scores were better, however there were multiple areas that could be improved. Overall, due to mentioned, the overall rating was 4, with recommendations to use only after significant changes of rigor of development. | Results, References |

# PRISMA 2020 Checklist

| Section and Topic | Item # | Checklist item | Location where item is reported |
|---|---|---|---|
| | | "Guidelines on The Management of Co-Occurring Alcohol and Other Drug and Mental Health Conditions in Alcohol and Other Drug Treatment Settings. Treatment Improvement Protocol TIP 42" developed by Substance Abuse and Mental Health Services Administration, which is branch of the U.S. Department of Health and Human Services and focuses on patients with co-occurring substance use disorders and mental health disorders. The scope and purpose of guideline were well described, except some minor information. The list of stakeholder involvement was impressively extensive, although there was no clear patient involvement. Some information on rigor of development could be improved to score with the highest standards. Clarity of presentation and applicability also had some rooms for improvement, however editorial independence was a concern given the nature of organization, funding, and competing interest issues. This resulted in overall rating of 5, with recommendation of using it with modifications.<br><br>"CANMAT Task Force Report on Cannabis Use", developed by CANMAT researchers, was focused on people with Bipolar Disorder, Major Depressive Disorder, and Cannabis Use. It scored robustly in methodology and rigor of development. However, information about external reviewing and updating were not provided. It scored moderately in stakeholder involvement and applicability domains, which supported the overall rating of 5, with recommendations to implement with modifications.<br><br>"SAMHSA Substance Use Disorder Treatment for People with Co-Occurring Disorders" was developed by SAMHSA focusing on people with co-occurring disorders. It focused on broad target of population, however, had one of the lowest scoring guidelines across all the domains. Overall rating was 3, with recommendation of use with modifications.<br><br>"ESTSS Recommendations for PTSD with Comorbid SUD" was focused on people with PTSD and SUD. It scored moderately in Scope and Purpose and Clarity. Otherwise, this guideline had multiple areas in different domains that could be improved to score better. Overall rating was 3, with the recommendation to be used after modifications.<br><br>"SAMHSA Treatment Considerations for Youth" by SAMHSA focused on youth and young adults and scored high in many domains, including rigor, clarity, applicability, however, editorial independence scores were very low. Overall rating was 5, however, the recommendation is to use with modifications and focus on possible bias due to unclear editorial independence.<br><br>"Australian Guidelines on Management of Co-Occurring Conditions" developed by Australian Government involvement, scored the highest overall score of 6, however still had weak Rigor of Development, and was recommended to use with possible modifications.<br><br>9. Neyra A, Parro-Torres C, Ros-Cucurull E, Carrera I, Echarri E, Torrens M. Management of schizophrenia and comorbid substance use disorders: expert review and guidance. Ann Gen Psychiatry. 2024 Oct 30;23(1):40. doi: 10.1186/s12991-024-00529-7. PMID: 39478536; PMCID: PMC11526640.<br>10. Spencer AE, Valentine SE, Sikov J, Yule AM, Hsu H, Hallett E, Xuan Z, Silverstein M, Fortuna L. Principles of Care for Young Adults with Co-Occurring Psychiatric and Substance Use Disorders. Pediatrics. 2021 Jan;147(Suppl 2):229-239. doi: 10.1542/peds.2020-023523F. PMID: 33386320; PMCID: PMC8276159.<br>11. Substance Use Disorder Treatment for People with Co-Occurring Disorders: Updated 2020 [Internet]. Rockville (MD): Substance Abuse and Mental Health Services Administration (US); 2020. (Treatment Improvement Protocol (TIP) Series, No. 42.) Available from: https://www.ncbi.nlm.nih.gov/books/NBK571020/.<br>12. Tourjman SV, Buck G, Jutras-Aswad D, Khullar A, McInerney S, Saraf G, Pinto JV, Potvin S, Poulin MJ, Frey BN, Kennedy SH, Lam RW, MacQueen G, Milev R, Parikh SV, Ravindran A, McIntyre RS, Schaffer A, Taylor VH, van Ameringen M, Yatham LN, Beaulieu S. Canadian Network for Mood and Anxiety Treatments (CANMAT) Task Force Report: A Systematic Review and Recommendations of Cannabis use in Bipolar Disorder and Major Depressive Disorder. Can J Psychiatry. 2023 May;68(5):299-311. doi: 10.1177/07067437221099769. Epub 2022 Jun 16. PMID: 35711159; PMCID: PMC10192829.<br>13. Substance Use Disorder Treatment for People with Co-Occurring Disorders: Updated 2020 [Internet]. Rockville (MD): Substance Abuse and Mental Health Services Administration (US); 2020. (Treatment Improvement Protocol (TIP) Series, No. 42.) Available from: https://www.ncbi.nlm.nih.gov/books/NBK571020/. | |

| Section and Topic | Item # | Checklist item | Location where item is reported |
|---|---|---|---|
| | | 14. Roberts NP, Lotzin A, Schäfer I. Psychological treatment of PTSD with comorbid substance use disorder (SUD): expert recommendations of the European Society for Traumatic Stress Studies (ESTSS). Eur J Psychotraumatol. 2023;14(2):2265773. doi: 10.1080/20008066.2023.2265773. Epub 2023 Oct 13. PMID: 37830207; PMCID: PMC10578096.<br>15. Substance Abuse and Mental Health Services Administration (SAMHSA): Treatment Considerations for Youth and Young Adults with Serious Emotional Disturbances/Serious Mental Illnesses and Co-occurring Substance Use. Publication No. PEP20-06-02-001. Rockville, MD: National Mental Health and Substance Use Policy Laboratory, Substance Abuse and Mental Health Services Administration, 2021.<br>16. Marel C, Siedlecka E, Fisher A, Gournay K, Deady M, Baker A, Kay-Lambkin F, Teesson M, Baillie A, Mills KL. Guidelines on the management of co-occurring alcohol and other drug and mental health conditions in alcohol and other drug treatment settings (3rd edition). Sydney, Australia: Matilda Centre for Research in Mental Health and Substance Use, The University of Sydney. 2022. | |
| Risk of bias in studies | 18 | Present assessments of risk of bias for each included study.<br>For the appraisal of guidelines the AGREE II (Appraisal of Guidelines for REsearch and Evaluation) tool was used independently by two reviewers, and if any disagreement or no consensus, a third reviewer, and eventually supervisor advised, | Results |
| Results of individual studies | 19 | For all outcomes, present, for each study: (a) summary statistics for each group (where appropriate) and (b) an effect estimate and its precision (e.g. confidence/credible interval), ideally using structured tables or plots.<br><br>Table 2. Full version of the AGREE II instrument (Strongly Disagree—1, Strongly Agree—7). | Results |

| GUIDELINES (Please See Table 1: Included Guidelines) | 1 | 2 | 3 | 4 | 5 | 6 | 7 | 8 |
|---|---|---|---|---|---|---|---|---|
| **DOMAIN 1. SCOPE AND PURPOSE** | | | | | | | | |
| 1. The overall objective(s) of the guideline is (are) specifically described. | 5 | 5 | 6 | 6 | 5 | 3 | 6 | 6 |
| 2. The health question(s) covered by the guideline is (are) specifically described. | 5 | 6 | 6 | 5 | 5 | 5 | 5 | 6 |
| 3. The population (patients, public, etc.) to whom the guideline is meant to apply is specifically described. | 2 | 4 | 5 | 5 | 3 | 3 | 6 | 7 |
| **DOMAIN 2. STAKEHOLDER INVOLVEMENT** | | | | | | | | |
| 4. The guideline development group includes individuals from all relevant professional groups. | 2 | 4 | 7 | 6 | 2 | 4 | 5 | 6 |
| 5. The views and preferences of the target population (patients, public, etc.) have been sought. | 2 | 1 | 2 | 5 | 2 | 3 | 4 | 6 |
| 6. The target users of the guideline are clearly defined. | 1 | 2 | 7 | 3 | 3 | 2 | 6 | 6 |
| **DOMAIN 3. RIGOR OF DEVELOPMENT** | | | | | | | | |
| 7. Systematic methods were used to search for evidence. | 6 | 2 | 2 | 6 | 1 | 3 | 6 | 2 |
| 8. The criteria for selecting the evidence are clearly described. | 4 | 1 | 1 | 6 | 1 | 4 | 6 | 1 |
| 9. The strength and limitations of the body of evidence are clearly described. | 1 | 2 | 1 | 7 | 1 | 4 | 5 | 4 |
| 10. The methods for formulating the recommendations are clearly described. | 1 | 1 | 4 | 7 | 1 | 2 | 6 | 5 |
| 11. The health benefits, side effects, and risks have been considered in formulating the recommendations. | 4 | 3 | 6 | 6 | 2 | 3 | 4 | 4 |
| 12. There is an explicit link between the recommendations and the supporting evidence. | 3 | 6 | 5 | 6 | 4 | 6 | 6 | 6 |
| 13. The guideline has been externally reviewed by experts prior to its publication. | 1 | 1 | 4 | 1 | 1 | 1 | 1 | 1 |
| 14. A procedure for updating the guideline is provided. | 1 | 1 | 2 | 1 | 1 | 1 | 1 | 2 |
| **DOMAIN 4. CLARITY OF PRESENTATION** | | | | | | | | |
| 15. The recommendations are specific and unambiguous. | 3 | 5 | 6 | 6 | 5 | 6 | 6 | 7 |
| 16. The different options for management of the condition or health issue are clearly presented. | 3 | 2 | 5 | 6 | 4 | 4 | 6 | 7 |
| 17. Key recommendations are easily identifiable. | 2 | 3 | 6 | 5 | 6 | 4 | 6 | 7 |
| **DOMAIN 5. APPLICABILITY** | | | | | | | | |
| 18. The guideline describes facilitators and barriers to its application. | 2 | 4 | 3 | 6 | 5 | 3 | 6 | 6 |
| 19. The guideline provides advice and/or tools on how the recommendations can be put into practice. | 4 | 5 | 6 | 6 | 4 | 2 | 5 | 6 |
| 20. The potential resource implications of applying the recommendations have been considered. | 2 | 4 | 3 | 2 | 4 | 2 | 6 | 5 |
| 21. The guideline presents monitoring and/or auditing criteria. | 1 | 1 | 2 | 1 | 1 | 1 | 5 | 5 |
| **DOMAIN 6. EDITORIAL INDEPENDENCE** | | | | | | | | |
| 22. The views of the funding body have not influenced the content of the guideline. | 2 | 5 | 1 | 3 | 2 | 1 | 2 | 4 |
| 23. Competing interests of guideline development group members have been recorded and addressed. | 4 | 6 | 1 | 4 | 1 | 3 | 2 | 1 |
| 1. Rate the overall quality of this guideline.<br>Lowest possible quality—1<br>Highest possible quality—7 | 3 | 4 | 5 | 5 | 3 | 3 | 5 | 6 |
| OVERALL CALCULATED BY DOMAIN AVERAGE | | | | | | | | |
| 2. I would recommend this guideline for use.<br>Yes 1, Yes with Modifications 2, No 3 | 2 | 2 | 2 | 2 | 2 | 2 | 2 | 2 |
| NOTES | | | | | | | | |

# PRISMA 2020 Checklist

| Section and Topic | Item # | Checklist item | Location where item is reported |
|---|---|---|---|
| | | | |
| Results of syntheses | 20a | For each synthesis, briefly summarise the characteristics and risk of bias among contributing studies. | |

**Table 3.** Short version of the Agree II instrument (Strongly Disagree—1, Strongly Agree—7).

| GUIDELINES (Please See Table 1: Included Guidelines) | 1 | 2 | 3 | 4 | 5 | 6 | 7 | 8 |
|---|---|---|---|---|---|---|---|---|
| DOMAIN 1. SCOPE AND PURPOSE | 4 | 5 | 6 | 5 | 4 | 4 | 6 | 6 |
| DOMAIN 2. STAKEHOLDER INVOLVEMENT | 2 | 2 | 5 | 5 | 2 | 3 | 5 | 6 |
| DOMAIN 3. RIGOR OF DEVELOPMENT | 3 | 2 | 3 | 6 | 2 | 3 | 6 | 4 |
| DOMAIN 4. CLARITY OF PRESENTATION | 3 | 3 | 6 | 6 | 5 | 5 | 6 | 7 |
| DOMAIN 5. APPLICABILITY | 2 | 4 | 4 | 4 | 4 | 2 | 6 | 6 |
| DOMAIN 6. EDITORIAL INDEPENDENCE | 3 | 6 | 1 | 4 | 2 | 2 | 2 | 3 |
| 1. Rate the overall quality of this guideline.<br>Lowest possible quality—1<br>Highest possible quality—7 | 3 | 4 | 5 | 5 | 3 | 3 | 5 | 6 |
| 2. I would recommend this guideline for use.<br>Yes—1, Yes with Modifications—2, No—3 | 2 | 2 | 2 | 2 | 2 | 2 | 2 | 2 |
| NOTES | | | | | | | | |

| Section and Topic | Item # | Checklist item | Location where item is reported |
|---|---|---|---|
| | 20b | Present results of all statistical syntheses conducted. If meta-analysis was done, present for each the summary estimate and its precision (e.g. confidence/credible interval) and measures of statistical heterogeneity. If comparing groups, describe the direction of the effect.<br><br>N/A | Results |
| | 20c | Present results of all investigations of possible causes of heterogeneity among study results.<br><br>N/A | Results |
| | 20d | Present results of all sensitivity analyses conducted to assess the robustness of the synthesized results. | Results |

# PRISMA 2020 Checklist

| Section and Topic | Item # | Checklist item | Location where item is reported |
|---|---|---|---|
| | | N/A | |
| Reporting biases | 21 | Present assessments of risk of bias due to missing results (arising from reporting biases) for each synthesis assessed.<br><br>N/A | Results |
| Certainty of evidence | 22 | Present assessments of certainty (or confidence) in the body of evidence for each outcome assessed.<br><br>N/A | Results |
| **DISCUSSION** | | | |
| Discussion | 23a | Provide a general interpretation of the results in the context of other evidence.<br><br>This review update addressed new concurrent disorder English language guidelines developed after our initial systematic review including guidelines from 2000 to 2020. Multiple new guidelines have been developed since 2020. We have included for the appraisal eight guidelines that met the criteria of being a formal clinical recommendation guideline with the primary focus on concurrent mental health and substance use disorders. Although there are multiple guidance and recommendations developed in the last five years, suggesting an increasing trend or recognition of importance, there still is a significant lack of availability of very specific focused, high quality concurrent disorder guidelines in different countries. All guidelines struggled with a limited evidence base, at times they did not include systematic evidence search and overall, there was a lack of rigor of even in the newer guidelines.<br><br>Sequential, parallel, and integrated models were as previously described levels of organization of integration of care. Integrated treatment models are considered to provide the best outcomes and are most cost effective, while offering one team providing addiction and mental health services within the same setting. Still, there is a need for more guidance and structure. Currently, the simple four quadrant model still is one of the few guidance's for infrastructure needs.<br>In order to address the level of care needs, we need conceptual agreed upon standards. This can be in the form of staging, such as used for cancer treatment, which allows to operationalize meaningfully guide to the level and type of care (e.g. stepped care). Measurement based care may help to more towards a personalized care. There are other function or risk-based models, but none seemed to have been acknowledged in the guidelines.<br><br>Based on evidence, the gold standard of concurrent disorder management suggests that with integrated care better outcomes of concurrent disorder management can be achieved, while their presence continue to be extremely rare in different health settings. Grading, staging, and functional level of an individual undergoing management are still not addressed. | Discussion |
| | 23b | Discuss any limitations of the evidence included in the review.<br><br>As in our initial search, all guidelines were ICD/DSM based. Guidelines also focused on specific combinations of disorders. Newer approaches such as the HiTOP model was not utilized any formulation of concurrent disorders in guidelines. Given the lack of diagnostic recommendation from ICD/DSM, most guidelines struggle with the complexity and diversity of dual disorders.<br><br>Similar to our previous review, the specific evidence for the comprehensive management of concurrent disorders is not available and only very specific questions have well-developed guidance. Some studies in concurrent disorder patients indicate that certain approaches working in individual disorders are less or not effective in concurrent disorders, such as SSRIs in alcohol-dependent individuals with major depressive disorder. There was some evidence of clozapine working better individuals with substance use disorder and schizophrenia. | Discussion |

# PRISMA 2020 Checklist

| Section and Topic | Item # | Checklist item | Location where item is reported |
|---|---|---|---|
| | | | |
| | 23c | Discuss any limitations of the review processes used.<br><br>This review is based on English language guidelines only. | Discussion |
| | 23d | Discuss implications of the results for practice, policy, and future research.<br><br>Overall, this updated review of current guidelines revealed consistent lack of evidence base in concurrent disorder, methodological gaps, lack of rigor in development and management recommendations which could be improved significantly. Furthermore, in any guideline, essential aspects for treatment planning, such as the concurrent disorder framework specifics, treatment needs “matching”, and the evaluation or severity “staging”, are not addressed. Development of guidelines that include patient perspectives, consideration of resource implications, more research in integrated. | Discussion |
| **OTHER INFORMATION** | | | |
| Registration and protocol | 24a | Provide registration information for the review, including register name and registration number, or state that the review was not registered.<br><br>The protocol for this systematic review was prepared according to the PRISMA-P checklist. The review was registered in the international register—PROSPERO (International Prospective Register of Ongoing Systematic Reviews, http://www.crd.york.ac.uk/prospero). The protocol of the study has been uploaded to PROSPERO.<br><br>Registration ID: CRD420251076197. | Introduction |
| | 24b | Indicate where the review protocol can be accessed, or state that a protocol was not prepared.<br><br>The protocol can be accessed at PROSPERO (https://www.crd.york.ac.uk/PROSPERO/view/CRD420251076197). | Introduction |
| | 24c | Describe and explain any amendments to information provided at registration or in the protocol.<br><br>The amendment was done to include all authors who were working on the review. | |
| Support | 25 | Describe sources of financial or non-financial support for the review, and the role of the funders or sponsors in the review.<br><br>**Funding:** This research received the following fundings:<br>1.S.H. as a Ph.D. student received previously a Four-Year Fellowship from The University of British Columbia Graduate and Postdoctoral Studies. This funding facilitated PhD studies.<br>2.The work was supported by World federation of Societies of Biological psychiatry (WFSBP) and an unrestricted fund from Richter Gedeon. There was no any direct influence or involvement of sponsors in creation of this work. | |
| Competing interests | 26 | Declare any competing interests of review authors. | |

PRISMA 2020 Checklist

| Section and Topic | Item # | Checklist item | Location where item is reported |
|---|---|---|---|
| | | **Conflict of Interest:** All authors declare no other conflicts of interest. | |
| Availability of data, code and other materials | 27 | Report which of the following are publicly available and where they can be found: template data collection forms; data extracted from included studies; data used for all analyses; analytic code; any other materials used in the review.<br><br>**Supplementary Materials:** The following supporting information can be downloaded at: https://www.mdpi.com/article/doi/s1<br><br>Data collection forms are available upon request. | |

*From:* Page MJ, McKenzie JE, Bossuyt PM, Boutron I, Hoffmann TC, Mulrow CD, et al. The PRISMA 2020 statement: an updated guideline for reporting systematic reviews. BMJ 2021;372:n71. doi: 10.1136/bmj.n71.
This work is licensed under CC BY 4.0. To view a copy of this license, visit https://creativecommons.org/licenses/by/4.0/ [21].
